# Clusterin expression and germinal center morphometry help distinguish idiopathic multicentric castleman disease from select lymphadenopathies

**DOI:** 10.3389/fimmu.2026.1872505

**Published:** 2026-09-17

**Authors:** Michael V. Gonzalez, Kaiwen Wang, Joseph Zinski, Melanie D. Mumau, Katherine S. Forsyth, Abiola H. Irvine, Joshua Brandstadter, Stacy G. Guzman, Lu Zhang, Sheila K. Pierson, Bridget Austin, David C. Fajgenbaum

**Affiliations:** 1 Center for Cytokine Storm Treatment & Laboratory, Department of Medicine, University of Pennsylvania, Philadelphia, PA, United States; 2 Division of Hematology/Oncology, Department of Medicine, Perelman School of Medicine, University of Pennsylvania, Philadelphia, Pennsylvania, PA, United States; 3 Department of Biochemistry, Biophysics, and Chemical Biology, University of Pennsylvania Perelman School of Medicine, Philadelphia, PA, United States; 4 Department of Hematology, Peking Union Medical College Hospital, Chinese Academy of Medical Sciences and Peking Union Medical College, Beijing, China

**Keywords:** bioinformatics, Clusterin (CLU), gene expression, idiopathic multicentric Castleman disease (iMCD), lymphadenopathy

## Abstract

Idiopathic multicentric Castleman disease (iMCD) is a heterogeneous cytokine storm disorder involving systemic inflammation, multicentric lymphadenopathy with characteristic histopathology, and life-threatening multiple organ dysfunction. Patients can present with symptoms ranging from thrombocytopenia, anasarca, fever/elevated C-reactive protein (CRP), reticulin myelofibrosis, renal dysfunction, and organomegaly (iMCD-TAFRO) to thrombocytosis, hypergammaglobulinemia, and plasmacytosis (iMCD-IPL), with patients not falling into either group (iMCD-NOS). The molecular mechanisms across the clinical subtypes have not been elucidated and new effective treatments are needed. Here, we performed bulk RNA sequencing and targeted gene expression quantification in lymph node tissue from multiple iMCD clinical subtypes, pathologically related disorders, and controls. We identified 249 upregulated and 42 downregulated genes in iMCD-TAFRO lymph node tissue, which were enriched in the following pathways: angiogenesis, cell proliferation, and various aspects of the humoral and innate immune response. The targeted gene expression analysis revealed shared differentially expressed genes (DEGs) across all three iMCD clinical subtypes, including *SPP1*, *XBP1*, *PRDM1*, and *CLU*. We identified unique DEGs in each iMCD clinical subtype, including *PLA2G2A, MSR1, IL8* (iMCD-TAFRO), *IL9, KIR3DL1, IL18RAP* (iMCD-IPL), and *IL7, TLR8, and CX3CR1* (iMCD-NOS). Since Clusterin (*CLU*) was significantly upregulated in iMCD but not in related conditions, we performed immunohistochemistry and observed that Clusterin is elevated in the germinal center and mantle zone regions of iMCD patient lymph nodes. A composite model incorporating CLU expression in these lymph node compartments with germinal center features demonstrated strong performance in differentiating iMCD from selected lymphadenopathies. Together, this work identified pathways dysregulated in iMCD lymph nodes and suggests that Clusterin, particularly when integrated with germinal center features, could be a novel biomarker.

## Introduction

Idiopathic multicentric Castleman Disease (iMCD) is a rare polyclonal lymphoproliferative disorder of unknown etiology with an annual incidence of approximately 1,500 individuals in the United States and a 25-35% 5-year mortality rate (1–3). iMCD is characterized by a wide range of clinical and pathological features, including multifocal lymphadenopathy with unique histopathologic features and cytokine-driven inflammation (4). Within iMCD, multiple clinical phenotypes exist (5). Patients presenting with thrombocytopenia, anasarca, fever/elevated C-reactive protein (CRP), reticulin myelofibrosis/renal dysfunction, and organomegaly have been defined as iMCD-TAFRO. iMCD with idiopathic plasmacytic lymphadenopathy (iMCD-IPL) is defined by thrombocytosis, hypergammaglobulinemia, and a milder clinical course. Patients who do not meet these clinical criteria are classified as iMCD–not otherwise specified (iMCD-NOS), which has milder clinical features more similar to iMCD-IPL than to iMCD-TAFRO. Patients can also be classified by histopathological subtype, including hyaline vascular/hypervascular, plasmacytic, and a mixed variant that incorporates features of both. However, the clinical implications of these histopathological subtypes remain unclear (6).

The etiology of iMCD is not well understood, and its heterogeneous presentation suggests multiple possible pathophysiological mechanisms. Roughly half of iMCD patients respond to siltuximab, a monoclonal antibody targeting the pro-inflammatory cytokine interleukin-6 (IL-6 (7, 8). Systemic inflammatory symptoms of iMCD can be observed in other inflammatory disorders and hematologic malignancies, complicating its diagnosis. However, gene expression differences in lymph node (LN) tissue between these clinically and pathologically related disorders have not been thoroughly explored, providing fertile ground to better identify more specific features of iMCD.

Most discovery-based investigations of gene expression networks or proteomic signatures involved in iMCD have been done in circulating peripheral blood mononuclear cells (PBMCs) or in plasma/serum, but not in the LN (8–11). Nevertheless, important pathogenic insights have been made. In a large serum proteomics-based study involving 88 iMCD patients and several clinico-pathologically related disorders, we found that the PI3K-AKT-mTOR, angiogenesis, IL-6-JAK-STAT3, and CXCL13 pathways, as well as up-regulated cytokines, were among the most enriched in iMCD serum (3, 8, 9). In PBMCs, we found that interferon-stimulated genes (ISGs) were upregulated and strongly correlated with mTOR signaling in several immune cell types in iMCD patients (10); interestingly, mTOR inhibition is an effective treatment in some patients. More recently, Wing et al. (12) and Horna et al. (13) explored gene expression changes in MCD LN tissue compared to healthy controls. These studies identified plasma cell differentiation (*XBP1*), follicular dendritic cell markers (*CXCL13*, *CLU*), angiogenesis (*VEGF, VEGFR*), mTORC1 pathway genes, and complement cascade signatures that were significantly increased in MCD LN tissues (12, 13). However, the sample size was limited in these studies, and HHV-8 status was not defined in Wing et al. Additionally, subtyping based on the clinical manifestations of MCD was not performed in either study.

Herein, we investigate the LN transcriptome across multiple iMCD subtypes (iMCD-TAFRO, iMCD-IPL, iMCD-NOS) compared to both sentinel controls and clinico-pathologically overlapping autoimmune (systemic lupus erythematosus, SLE) and neoplastic (diffuse large B-cell lymphoma, DLBCL) conditions, and perform targeted studies of genes that distinguish these conditions. Although these subtypes of iMCD have been defined and described (14, 15), the LN transcriptomic differences have not been explored.

## Materials and methods

### Patient samples and histologic review

#### Discovery and validation cohorts

We identified samples from 7 patients enrolled in the ACCELERATE natural history registry (NCT02817997) that met criteria for iMCD according to current international diagnostic criteria (16). Additionally, all samples/studies described were approved by the Institutional Review Board of the University of Pennsylvania (IRB # 824758). Based on clinical and laboratory data collected at the time of diagnosis, patients were categorized as iMCD-TAFRO (n=7) (17). Additionally, 7 LN samples resected from breast cancer patients negative for metastasis were chosen to represent controls, herein referred to as sentinel controls (**Table 1**). Histologically negative sentinel lymph nodes were used as a control group because they represent non-diseased lymphoid tissue obtained through routine clinical care that lack the clinical and pathological features of iMCD and other lymphadenopathies under study. We also obtained LN samples from patients with DLBCL (n=5) from a University of Pennsylvania pathology core service.

**Table 1:** Demographic and clinical characteristics of study cohorts.

Characteristic	Discovery: sentinel controls (n=7)	Discovery: iMCD-TAFRO (n=7)	Nanostring: sentinel controls (n=8)	Nanostring: iMCD-TAFRO (n=12*)	Nanostring: iMCD-IPL (n=3)	Nanostring: iMCD-NOS (n=4)	Nanostring: SLE (n=4)	Nanostring: DLBCL (n=5)
Age, mean (SD)	NA	49.1 (12.01)	NA	44.7 (13.71)	40.9 (12.07)	44.4 (16.67)	NA	NA
Sex (M:F)	NA	2:5	NA	4:8	1:2	3:1	NA	NA
Location of tissue sampled, n (%)	NA	Axillary 4 (67%), Groin 1 (16.7%), Mediastinal 1 (16.7%), Not specified 1 (16.7%)	NA	Axillary 5 (41.7%), Inguinal 2 (16.7%), Cervical 1 (8.3%), Groin 1 (8.3%), Mediastinal 1 (8.3%)	Cervical 2 (66%), Groin 1 (33%)	Axillary 2 (50%), Abdominal 1 (25%), Cervical 1 (25%)	NA	NA
Hyaline vascular, n (%)	NA	5 (83.3%)	NA	9 (75%)	1 (33%)	2 (50%)	NA	NA
Plasmacytic or mixed, n (%)	NA	1 (16.7%)	NA	3 (25%)	2 (66%)	2 (50%)	NA	NA
Not specified histology, n (%)	NA	1 (16.7%)						

NA, Not Available; SD, Standard Deviation. *7 iMCD-TAFRO samples in the Nanostring cohort overlap bulk RNAseq samples described in the Discovery cohort.

In addition to the discovery cohort, we identified 22 additional patients enrolled in the ACCELERATE natural history registry. These patients were categorized into two subtype groups in the validation cohort: iMCD-TAFRO (n=15) and iMCD-NOS/IPL (n=12). The NOS/IPL subtypes were combined because the number of IPL samples was insufficient for comparison (n=1). We also included previously published iMCD samples and sentinel controls (15), which included 8 non-specific MCD and 19 sentinel controls. The composition of the validation cohort is summarized in **Supplementary Table 1**.

#### Targeted gene expression cohort

Finally, our targeted gene expression cohort included multiple iMCD clinical subtypes and inflammatory disease comparators. Based on clinical and laboratory data collected at the time of diagnosis, patients were categorized into one of three subtypes: iMCD-TAFRO (n=12), iMCD-IPL (n=3), or iMCD-NOS (n=4) (18). In addition to the iMCD subtype samples, SLE (n=3) and DLBCL (n=5) samples were used as disease comparator groups (**Table 1**). RNA integrity values were used to select high-quality RNA samples. Samples that overlap between the Discovery and Targeted Gene Expression Cohort can be found in **Supplementary Table 2**.

### Sample preparation and gene expression quantification

#### RNA extraction from FFPE unstained slides

RNA was isolated from Formalin Fixed Paraffin Embedded (FFPE) tissue sections fixed on slides using the RNeasy DSP FFPE kit (Qiagen, Hilden, Germany). Briefly, sections 10 microns thick were scraped from 5 slides of each sample and submerged in Deparaffinization Solution (DSP) according to the protocol. During this process, tissue digestion using Proteinase K was also done. DNAseI-treated RNA was subsequently isolated using Qiagen’s proprietary spin column method, as per the protocol. RNA integrity was assessed using the TapeStation RNA ScreenTape (Agilent, Santa Clara, CA), and concentration was determined using the Qubit 2.0 Fluorometer (Thermo Fisher Scientific, Waltham, MA). Since FFPE RNA is expected to be highly degraded, a DV200 value, representing the percentage of RNA fragments greater than 200 bp, was determined for each sample. In the validation cohort, RNA sample preparation and short-read sequencing were performed as previously described (19).

#### Preparation of bulk RNA sequencing samples

Libraries for whole transcriptome RNA sequencing were prepared using the Stranded Total RNAseq with Ribo-zero Plus kit (Illumina, San Diego, CA) as per the manufacturer’s instructions, starting with an input of 100ng of RNA and 15 cycles of final PCR amplification. Library size was assessed using the 2100 Bioanalyzer and the High-Sensitivity DNA assay (Agilent, Santa Clara, CA). Concentration was determined using the Qubit Fluorometer 2.0. Next-generation sequencing with a paired-end 2x100 bp run length was performed on the NextSeq 2000 platform (Illumina, San Diego, CA). A minimum of 30M reads per sample was acquired for each sample.

#### Preparation of Nanostring nCounter samples

100 ng of RNA fragments 200 bp and over were used for the NanoString nCounter assay (NanoString, Seattle, WA). Briefly, RNA was hybridized at 65 °C for 19 hours with proprietary fluorescently labeled reporter and capture probes to bind target genes. The hybridized mix of probes and target genes was washed, enriched, and isolated using the NanoString Prep station, and then scanned for fluorescent intensity using the NanoString Digital Analyzer. The nCounter Immunology panel (v2, **Supplementary Table 3**) was used to quantify 594 genes known to be involved in autoimmune/immune responses, along with 15 internal reference control genes to facilitate normalization.

### Informatic and gene expression analysis

#### Bulk RNAseq analysis in discovery and validation cohorts

RNA-seq reads were demultiplexed using bcl2fastq (Illumina, San Diego). Demultiplexed FASTQ files were aligned to GRCh38 using the Kallisto pseudo-aligner (20) using default settings. The generated BAM files were read into the R statistical computing environment, and gene counts were quantified using the GenomicAlignments package (21). Preliminary QC and differential expression analysis were performed using the R/Bioconductor package DESeq2 (22). Read counts were filtered to include only genes with a read count >=20 in greater than 33% of samples. In the discovery cohort, patients were grouped by phenotype (iMCD-TAFRO, iMCD-NOS/IPL, DLBCL, and sentinel control), and DEGs were considered for further investigation using a log_2_ fold change (log_2_FC)>|1| and a Benjamini-Hochberg corrected *P*-value<0.05. Gene Set Enrichment analysis (23) (GSEA, v.4.3.2) was performed for the iMCD-TAFRO (n=7) vs. sentinel controls (n=7) discovery cohort using phenotype permutation settings. Gene sets were considered significant if the false discovery rate q-value < 0.25. The Hallmark gene sets from msigDB were used for analysis (24).

For validation, we utilized an expanded cohort comprising data from three sources, as described above. Batch correction was applied to the cohort’s normalized expression matrix using the limma package (25). The iMCD-TAFRO and sentinel control samples used in the discovery cohort analysis were removed to maintain independence between datasets. DEG analysis was performed using the same workflow and constraints as the discovery cohort.

#### Nanostring RNA nCounter assay

Reporter code counts (RCC) were read into R using NanostringQCPro (26). Quality control and normalization were performed using RUVSeq (27, 28). RUVSeq utilizes a panel of 15 housekeeping genes and is able to normalize based on their expression levels (*EEF1G, TUBB, TBP, POLR2A, GUSB, HPRT1, GAPDH, SDHA, OAZ1, PPIA, G6PD, RPL19, POLR1B, ABCF1, and ALAS1*). After normalization, DESeq2 was used to perform differential expression analysis, and DEGs were identified using the discovery cohort criteria.

#### Estimation and comparison of cell type composition in bulk RNAseq data

Single-cell RNA sequencing data from mesenteric and lung-draining LN tissue from 12 deceased organ donors were downloaded from the CellTypist Immune Atlas (Celltypist.org, accessed June 30, 2023 (29). Annotated cells organized into 13 cell types (B cells, Cycling cells, Dendritic cells, Erythroid, HSC/MPP, ILC, Macrophages, Mast cells, Monocytes, Plasma cells, Promyelocytes, T cells, pDCs) were used to deconvolute the iMCD bulk RNA sequencing samples into estimated cell type proportions using the R-based software tool, Bisque, using a non-negative least-squares regression model (30). Inferred cell proportions per cell type were used to perform composition-based comparisons between iMCD-TAFRO, iMCD-NOS/IPL, and sentinel control samples using a Wilcoxon rank sum test. A Bonferroni correction was used to account for multiple comparisons.

#### Querying LINCS1000 database to predict drug perturbation efficacy in iMCD TAFRO

L2S2 was used to predict potential reversing therapeutic perturbations (31). The top DEGs from the iMCD-TAFRO versus sentinel control LN comparison (± log_2_FC>1.5, adjusted-*P* < 0.05) were used to construct a gene signature for database entry, and standard settings were used to predict reversing drug perturbations. Predicted reversing drugs were grouped by mechanism of action (MOA) and were ranked by the number of significant individual drugs within that group. For each compound within each cell line, replicate P-values were collapsed into a single line-level value using the MODZ-style robust z-mean (p→z, Tukey biweight mean, z→p) previously employed (32). Line-level P-values for the same compound were then meta-analyzed across cell lines using Stouffer’s Z (equal weights), restricting to cell lines with P<0.05 to focus on lines showing significant reversal signal. Scores are reported as −log_10_P and used for ranking. We repeated the procedure after mapping compounds to MOA to obtain MOA-level scores. Results are presented in four contexts: all lines (“iMCD”), all lines restricted to immune cell lines only (“iMCD-Immune”), iMCD-TAFRO, and iMCD-TAFRO restricted to immune cell lines only (TAFRO-immune). Figures display the top hits by −log10P.

#### Quantification of follicular clusterin expression and germinal center features for ROC-based discrimination of iMCD from non-CD controls

To label Clusterin in LNs, we immunohistochemically stained for Clusterin using DAB (3,3′-diaminobenzidine) with a hematoxylin counterstain to mark nuclei. Samples overlapping between the previously described discovery and targeted NanoString cohorts and the IHC cohort are listed in **Supplementary Table 2**. We then identified a DAB threshold to divide Clusterin into positive and negative regions. To analyze the Clusterin DAB and nuclear hematoxylin stains, we developed a Python-based algorithm quantify Clusterin staining intensity. We used the rgb2hed function from the Python scikit-image library to separate the DAB and hematoxylin channels. We quantified DAB stain in optical density (OD) units. We identified the LN from the background using a global Otsu threshold applied to the LN on the hematoxylin channel. This LN segmentation was used to measure the total LN area. Germinal centers (GCs) and mantle zones (MZs) were manually segmented to attain representative samples. GC, MZ, and interfollicular (IF) regions were manually segmented using the hematoxylin channel alone to blind users to the Clusterin stain. For each sample, the total number of GCs was manually enumerated, using serial H&E sections as a reference for morphological features. We then used the total GC count to randomly identify 10 GCs and MZs for segmentation. To determine statistical significance for all plots, an ANOVA analysis was performed, followed by Tukey tests to compare all conditions. Compartment-resolved Clusterin intensity (IF, MZ, and GC), GC density, and mean GC size were integrated into a model to differentiate between iMCD (n = 20) and non-Castleman nodes (sentinel, SLE, DLBCL, and FDC sarcoma; n = 36) by ROC analysis (scikit-learn) by an L2-regularized logistic-regression model evaluated by repeated stratified 5-fold cross-validation with a nested bootstrap 95% CI and label-permutation significance. Because DLBCL and FDC sarcoma lack mantle zones and germinal centers, those features were set to zero for those cases.

## Results

### iMCD-TAFRO shows a distinct LN transcriptome profile compared to sentinel controls

Prior to investigating transcriptomic changes in iMCD, we compared DEGs between DLBCL (n=5) and sentinel control LN samples (n=7) with gene expression quantification from a DLBCL cohort of 414 individuals (33). Importantly, we observed several well-established DLBCL gene signatures in our cohort, including minichromosome maintenance complex genes (*MCM2, MCM3, MCM4*), collagen proteins (*COL8A1, COL9A2*), cytokine-mediated signaling and inflammatory genes (*CXCL9, CXCL10*), and interleukins and interleukin receptors (*IL17RB, IL1R2, IL21R, ILDR2*) (**Supplementary Table 4****).** The concordance between the DEGs identified in this cohort and published DLBCL literature supports the use of this assay to uncover established transcriptomic changes and explore novel aspects of iMCD biology.

Next, we applied our approach to iMCD-TAFRO, a homogeneous, phenotypically severe subtype of iMCD that has not been specifically characterized at the transcriptomic level. We performed bulk RNA sequencing on FFPE LN tissue from iMCD-TAFRO patients (n=7) and sentinel controls (n=7). Principal component analysis of the top 500 most variable genes revealed moderate separation between the two groups (**Supplementary Figure 1**), with PC1 and PC2—enriched with immune-related and cell signaling genes (**Supplementary Figures 2A, B**)—providing the clearest distinction between iMCD-TAFRO and sentinel control samples.

Differential expression analysis identified 249 genes significantly upregulated in iMCD-TAFRO compared to sentinel controls, and 42 genes were significantly downregulated. The three most up-regulated genes in iMCD-TAFRO were *SPP1*, a multifunctional gene with critical roles in lymphocyte chemotaxis and cytokine regulation, *PLA2G2A*, which is secreted by activated macrophages and platelets and can amplify inflammation, and *UTY*, thought to be involved in chromatin regulation and protein-protein interactions. *IL-6*, a driver of disease progression in some patients, was upregulated and showed a statistically significant increase (log_2_FC=1.83, adjusted-*P* = 0.002). Other notable upregulated genes included *CLU*, *THY1, CD93, LBP*, and *SERPINE1*, which are involved in diverse immune responses. Additionally, *POSTN, COL15A1, and COL21A1* contribute to tissue remodeling (**Figure 1**, **Supplementary Table 5**).

**Figure 1:**
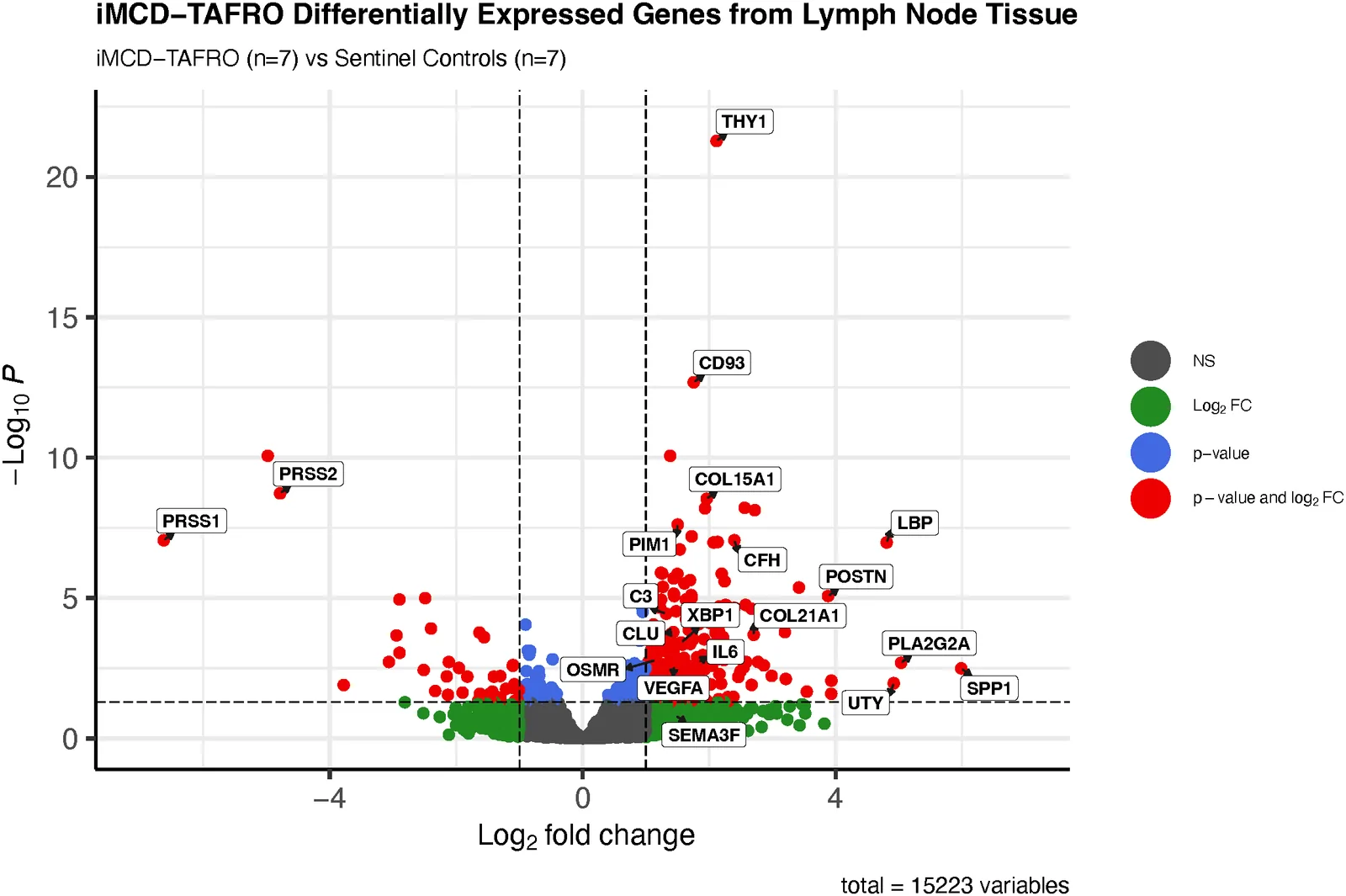
Differentially expressed genes in iMCD-TAFRO (n=7) vs sentinel control (n=7) lymph node tissue. A volcano plot of up- and down-regulated genes in iMCD-TAFRO vs sentinel control samples in the discovery cohort. Genes previously implicated in Castleman Disease have been labeled. Vertical dotted lines indicate a +/- log2 fold change (log2FC) threshold of 1, and horizontal dotted lines indicate a threshold for an adjusted P <0.05. Red circles indicate genes that pass both log2FC and adjusted P-value thresholds. Green circles indicate genes that pass the log2FC threshold, but do not have a significant adjusted P-value. Blue circles indicate genes that do not pass the log2FC threshold but have a significant adj. P-value. Grey circles are genes that do not pass either the log2FC or the adj. P-value thresholds.

We then performed gene set enrichment analyses to identify potential gene pathways involved in iMCD-TAFRO pathogenesis using the hallmark gene sets from mSigDB (24). 33 out of the 50 hallmark gene sets were significantly enriched in iMCD-TAFRO (Normalized enrichment score, NES>1.5, and FDR q-val<0.05), including pathways involved in immune signaling (e.g., *IL2-STAT5* signaling, *IL6-JAK-STAT3* signaling, *mTORC1* signaling), cellular remodeling (e.g., epithelial-mesenchymal transition, *MYC* targets, angiogenesis), and metabolic reprogramming pathways (e.g., oxidative phosphorylation, glycolysis) (**Table 2**). Several of these pathways, including angiogenesis and cellular proliferation, have been previously implicated in the pathogenesis of iMCD-TAFRO.

**Table 2:** Significantly enriched hallmark gene pathways in the transcriptome of iMCD-TAFRO lymph node (FDR <0.1, NES >1.5).

Description	Set size	NES	Adjusted p	FDR
EPITHELIAL_MESENCHYMAL_TRANSITION	179	2.98	6.25E-10	1.32E-10
COAGULATION*	98	2.75	6.25E-10	1.32E-10
ANGIOGENESIS	27	2.75	6.25E-10	1.32E-10
TNFA_SIGNALING_VIA_NFKB	183	2.66	6.25E-10	1.32E-10
MYC_TARGETS_V1	192	2.52	6.25E-10	1.32E-10
HYPOXIA	169	2.50	6.25E-10	1.32E-10
MTORC1_SIGNALING	190	2.45	6.25E-10	1.32E-10
INFLAMMATORY_RESPONSE	173	2.40	6.25E-10	1.32E-10
UNFOLDED_PROTEIN_RESPONSE	100	2.37	4.41E-09	9.29E-10
UV_RESPONSE_DN	138	2.26	4.41E-09	9.29E-10
APOPTOSIS*	147	2.20	4.41E-09	9.29E-10
ANDROGEN_RESPONSE	90	2.32	7.72E-09	1.63E-09
IL2_STAT5_SIGNALING	185	2.06	5.86E-08	1.23E-08
COMPLEMENT*	175	2.07	9.47E-08	1.99E-08
INTERFERON_GAMMA_RESPONSE	188	2.02	1.93E-07	4.06E-08
IL6_JAK_STAT3_SIGNALING	76	2.26	2.94E-07	6.19E-08
PROTEIN_SECRETION	91	2.19	3.80E-07	8.01E-08
KRAS_SIGNALING_UP	167	2.01	3.80E-07	8.01E-08
GLYCOLYSIS	171	1.98	5.37E-07	1.13E-07
OXIDATIVE_PHOSPHORYLATION	180	1.98	7.14E-07	1.50E-07
APICAL_JUNCTION	165	1.99	9.43E-07	1.99E-07
G2M_CHECKPOINT	184	1.91	1.88E-06	3.97E-07
ESTROGEN_RESPONSE_EARLY	172	1.93	2.61E-06	5.49E-07
CHOLESTEROL_HOMEOSTASIS*	66	2.02	2.70E-05	5.69E-06
MITOTIC_SPINDLE	195	1.80	4.69E-05	9.87E-06
ESTROGEN_RESPONSE_LATE	161	1.80	4.69E-05	9.87E-06
UV_RESPONSE_UP	134	1.86	8.66E-05	1.82E-05
ADIPOGENESIS	182	1.68	5.40E-04	1.14E-04
TGF_BETA_SIGNALING	53	1.92	5.83E-04	1.23E-04
E2F_TARGETS	190	1.64	6.21E-04	1.31E-04
P53_PATHWAY	176	1.62	1.66E-03	3.49E-04
HEDGEHOG_SIGNALING	30	1.90	1.75E-03	3.69E-04
MYOGENESIS*	148	1.64	1.75E-03	3.69E-04

*Hallmark gene pathways that include CLU.

### Upregulated transcriptomic changes validated in an independent iMCD-TAFRO cohort

To validate these findings, we performed transcriptomic analyses on an independent cohort of iMCD-TAFRO (n=15) and sentinel control samples (n=19), which were batch-corrected and normalized (**Supplementary Figure 3**). Of the 249 genes upregulated in the discovery cohort, 220 (88%) were also significantly upregulated in the validation cohort. The remaining 29 genes showed a similar directional trend but did not reach statistical significance. Consistent with this, the 20 most up-regulated genes in the discovery cohort retained their rank order and showed concordant gene expression changes in the validation cohort (**Table 3**, **Supplementary Table 5**). We also examined all 15,223 overlapping genes between the two analyses and observed strong concordance across the two datasets; 13,169 (86.5%) exhibited the same direction of change in both DEG analyses (**Supplementary Figure 4****;** Pearson correlation coefficient of 0.86, p<2.2×10^-16^). Together, these results validate the findings in our discovery cohort.

**Table 3:** Top 20 genes by log_2_ fold change in the discovery iMCD-TAFRO cohort, with corresponding validation cohort statistics, adjusted p-values, and ranks in both cohorts.

Gene	log2FC (validation)	log2FC (discovery)	Adjusted p (validation)	Adjusted P (discovery)	Rank (validation)	Rank (discovery)
*SPP1*	7.62	5.99	3.50E-17	3.21E-03	1	1
*PLA2G2A*	3.10	5.04	2.15E-03	2.02E-03	15	2
*UTY*	4.21	4.92	2.46E-02	1.08E-02	11	3
*LBP*	6.37	4.81	6.08E-12	1.05E-07	2	4
*MGAM*	5.21	3.93	1.19E-12	8.79E-03	4	5
*AQP9*	4.93	3.93	1.72E-12	2.58E-02	7	6
*POSTN*	2.87	3.88	4.01E-07	8.53E-06	16	7
*SLC19A2*	2.54	3.82	1.10E-14	3.00E-01	18	8
*INHBB*	3.41	3.54	1.03E-10	2.17E-02	14	9
*HLA-DQA2*	5.04	3.52	4.50E-06	1.29E-01	5	10
*STON1-GTF2A1L*	2.34	3.51	2.65E-04	6.79E-02	19	11
*IGKV3D-15*	3.84	3.47	8.03E-07	6.08E-02	12	12
*STS*	4.73	3.43	3.04E-05	3.42E-01	9	13
*PLPPR4*	3.79	3.42	2.62E-14	4.23E-06	13	14
*NUDCD1*	5.33	3.27	1.88E-24	7.20E-02	3	15
*IGLV2-18*	1.46	3.24	2.85E-02	2.17E-01	20	16
*S100A8*	4.33	3.21	1.73E-11	7.61E-03	10	17
*SERPINE1*	2.76	3.20	4.21E-05	1.64E-04	17	18
*RP11-561B11.2*	4.74	3.07	7.43E-11	1.29E-01	8	19
*HSPA5*	4.98	3.05	1.35E-04	7.11E-02	6	20

### Transcriptomic signatures reveal differential cell type enrichment and potential repurposed treatments

After validating the transcriptomic changes across independent cohorts, we evaluated gene expression in the omnibus bulk RNA-seq cohort (iMCD=37, Sentinel controls=26) across the clinically defined subtypes (iMCD-TAFRO=23, iMCD-IPL/NOS=14) to infer changes in cell type proportions and make treatment predictions. We performed cell type deconvolution to estimate cell type proportions from normalized read count data and identified significantly higher proportions of monocytes, erythroid cells, plasma cells, and innate lymphoid cells, as well as a significantly lower number of dendritic cells, in iMCD-TAFRO compared to sentinel controls (**Figure 2A**). Our data also suggest significantly more monocytes and innate lymphoid cells and significantly fewer dendritic cells in iMCD-IPL/NOS compared to controls (**Figures 2B, C**).

**Figure 2:**
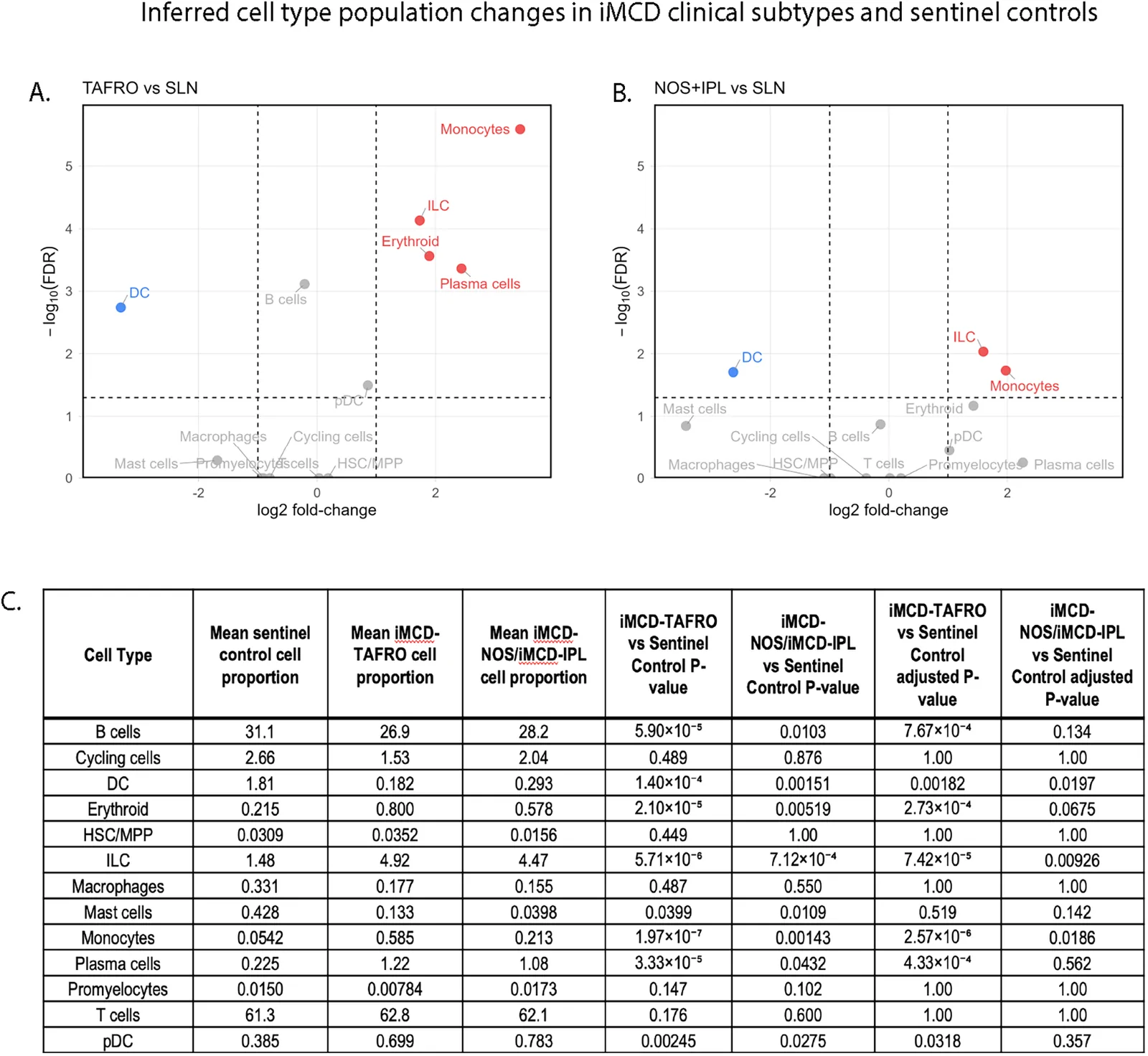
Inferred cell type population changes in iMCD clinical subtypes and sentinel controls. Cell types predicted to be significantly up- or down-regulated in **(A)** iMCD-TAFRO vs. SLN or **(B)** iMCD-NOS/IPL vs SLN. **(C)** Shows all cell type proportion predictions. dendritic cells (DC), innate lymphoid cells (ILC), Plasmacytoid Dendritic Cells (pDCs).

We identified potential treatment approaches that could be used in iMCD by examining both iMCD (all subtype) and iMCD-TAFRO gene signatures using the LINCS1000 database. DEGs with a log_2_FC greater than |1.5| from each comparison were used to identify predicted reversing drug treatments (**Figures 3A–F**, **Supplementary Tables 6**-**8**). Interestingly, sirolimus, a known treatment in iMCD, emerged as a top choice for reversal of the iMCD (all subtype) and iMCD-TAFRO gene signatures (**Figures 3A, B, D**, **Supplementary Table 7**). When DEGs from the TAFRO subtype were considered, interesting new potential new drugs emerge such as the VEGF inhibitor, sorafenib (**Figure 3C**, **Supplementary Table 8**).

**Figure 3:**
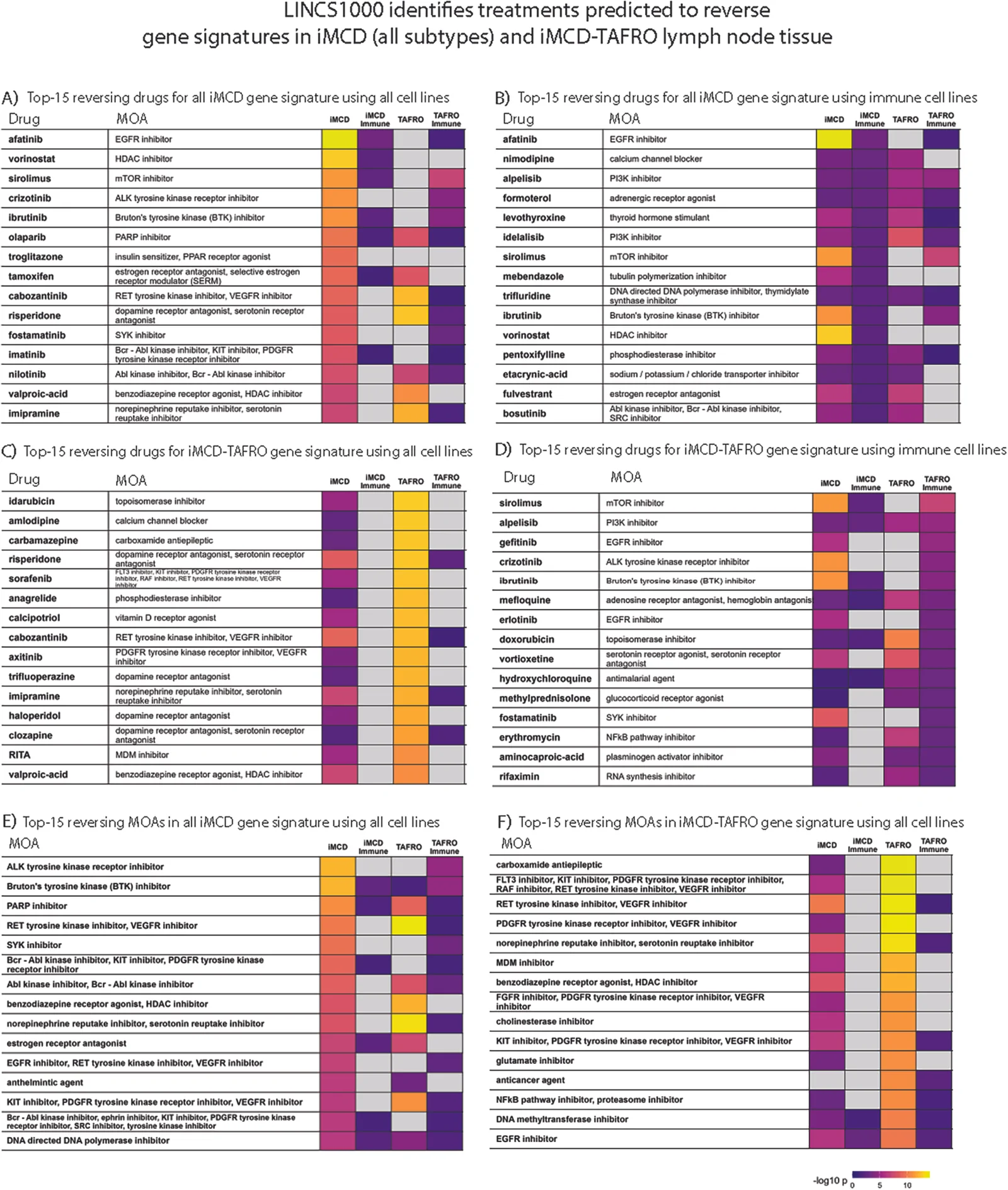
LINCS1000 identifies treatments predicted to reverse gene signatures in iMCD (all subtypes) and iMCD-TAFRO lymph node tissue. The heatmap encodes the LINCS1000 enrichment as −log10(p_global). Grey indicates that no cell lines were tested for that MOA or drug. Each row lists a Drug (bold) with its mechanism of action (MOA). **(A–D)** are scores for iMCD genes (all cell lines), iMCD genes (immune-restricted cell lines), TAFRO genes (all cell lines), and TAFRO genes (immune-restricted cell lines). **(A–D)** are sorted by these columns to display the top 15 for each. **(E, F)** aggregate drugs by MOA, displaying the top 15 MOAs ranked by median −log_10_(p_global) for iMCD and TAFRO gene signatures. 5-HT, serotonin; ACh, acetylcholine; AChE, acetylcholinesterase; act., activator; ag., agonist; aggr., aggregation; antag., antagonist; blk., blocker; COX, cyclooxygenase; DA, dopamine; DH, dehydrogenase; immunosupp., immunosuppressant; inh., inhibitor; mod., modulator; MTase, methyltransferase; NE, norepinephrine; PDE, phosphodiesterase; pol., polymerase; prod., production; rec., receptor; repl., replication; reup., reuptake; stim., stimulant; synth., synthesis; TK, tyrosine kinase; topo., topoisomerase; transp., transporter.

### Orthogonal gene expression analysis of iMCD subtypes shows shared and unique gene signatures

To determine unique and shared gene signatures between iMCD clinical subtypes and clinicopathologically related disorders, we performed targeted transcriptomic analysis of 594 immune-related genes using an orthogonal targeted gene expression platform (Nanostring nCounter) on iMCD-TAFRO (n=12), iMCD-NOS (n=4), iMCD-IPL (n=3), sentinel controls (n=8), SLE (n=3), and DLBCL (n=5) (**Table 1**). We then compared the DEGs identified using this method with those from the discovery bulk RNA-seq analysis and found that, among the 468 overlapping genes, the direction of association and fold change were highly correlated between the two methods (**Supplementary Figure 5**).

As expected, some DEGs were upregulated in all iMCD subtypes; however, each clinical subtype also exhibited unique gene expression signatures. Four DEGs were significantly upregulated across all clinical subtypes (*SPP1*, *PRDM1*, *XBP1*, and *CLU*; **Figure 4**, **Supplementary Table 11**) and were also significantly upregulated in the bulk RNA-seq iMCD-TAFRO data. Notably, *CLU* was the only gene uniformly upregulated across the three iMCD clinical subtypes and was not significantly upregulated in either of the clinicopathologically related disorders.

**Figure 4:**
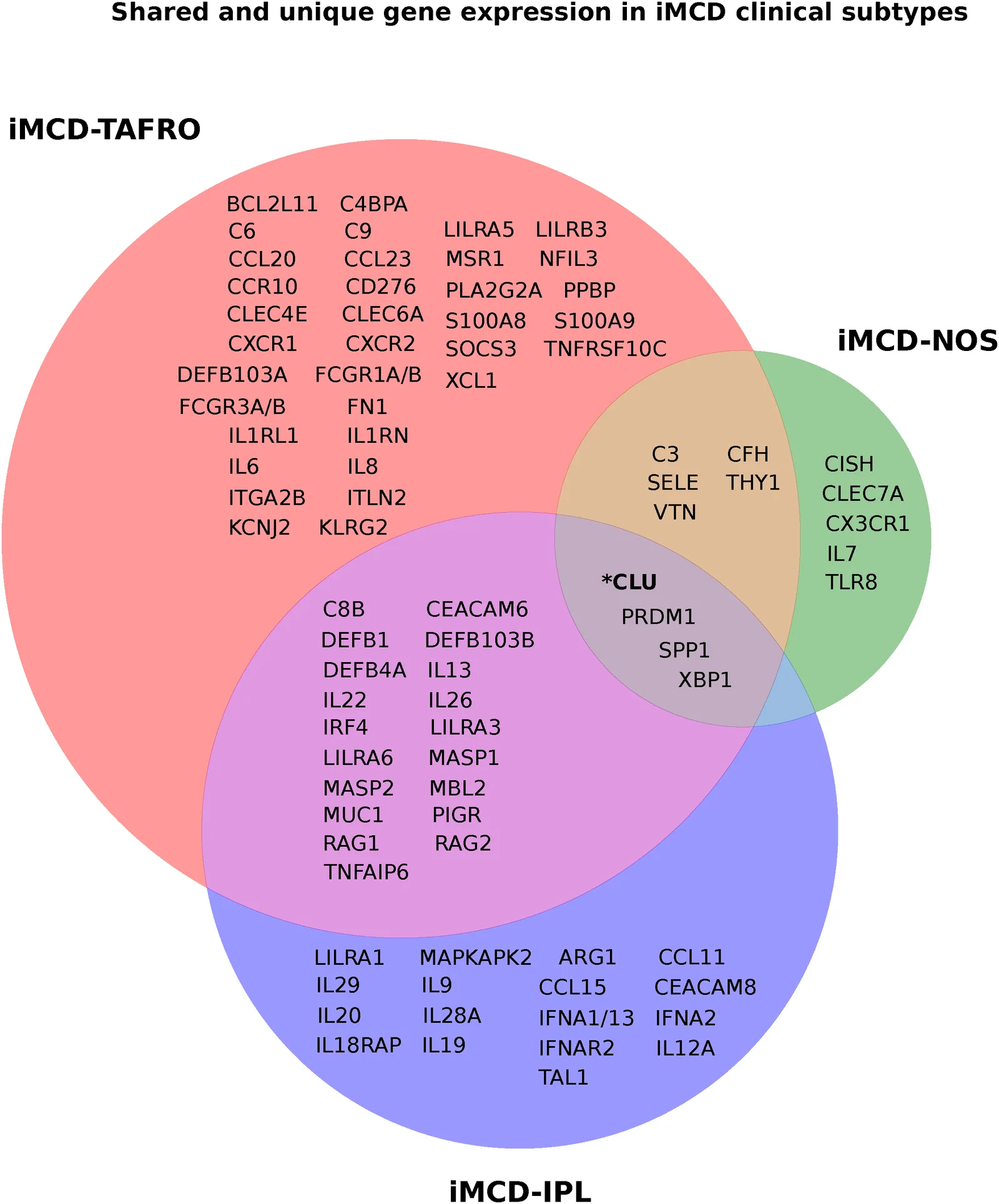
Shared and unique gene expression in iMCD clinical subtypes. Shared and unique genes in the targeted Nanostring gene expression panel in each iMCD clinical subtype. CLU was the only gene that was significantly upregulated in all 3 clinical subtypes, but not in any of the disease comparators.

There were also genes that exhibited subset-specific differential expression. There were 44 unique DEGs in iMCD-TAFRO (35 upregulated, 9 downregulated), 45 unique DEGs in iMCD-IPL (17 upregulated, 28 downregulated), and 12 unique DEGs in iMCD-NOS (5 upregulated, 7 downregulated (**Figure 4**, **Supplementary Tables 11**-**13**). The top unique DEGs in iMCD-TAFRO were *PLA2G2A*, *MSR1*, and *IL8*, in MCD-IPL were *IL9*, *KIR3DL1*, and *IL18RAP*, and in iMCD-NOS were *IL7*, *TLR8*, and *CX3CR1* (**Supplementary Tables 11**-**13**).

### Clusterin expression in regions of iMCD LN tissue and germinal center features differentiate iMCD from select lymphadenopathies

*CLU* was the only gene uniformly upregulated in iMCD clinical subtypes but not in any disease comparator groups. Furthermore, it is known as a marker for follicular dendritic cells (FDCs), a fibroblastic lymph node stromal cell known whose histologic prominence is a defining pathologic feature of Castleman. Markers such as CR1, CR2, FCER2, LTBR, FDCSP, and VCAM1 can help identify expanded follicular dendritic cell networks in Castleman disease. Importantly, CLU expression showed only modest and variable correlation with these FDC-associated genes, with stronger correlation for select markers such as FDCSP and VCAM1 but weak or absent correlation with canonical markers, including CR1, CR2, FCER2, and LTBR (**Supplementary Table 14**). These findings suggest that CLU expression may not simply reflect broad FDC enrichment. However, further studies will be required to determine whether CLU provides additive diagnostic or biological information beyond established FDC-associated markers and whether it represents a disease-specific biomarker in iMCD.

We therefore asked if *CLU* could distinguish iMCD from related diseases by single-plex immunohistochemistry, a widely used tool in pathologic diagnosis. We performed immunohistochemistry using the Clusterin protein, which is recognized by a robust and reproducible IHC. We quantified the area of positive Clusterin staining out of the total LN area in iMCD (n=20), two overlapping conditions (DLBCL, n=10, SLE, n=10), a condition known to have increased Clusterin (FDC sarcoma, n=6), and sentinel controls (n=10). As expected, FDC sarcoma had significantly increased Clusterin staining across the entire LN (**Figure 5A**). Next, we evaluated whether there were differences in Clusterin staining in different regions of the LN between disease groups. iMCD was the only disease group with significantly increased Clusterin staining in the MZ and the GC compared to sentinel LNs (**Figures 5B, D**). However, in the IF space, FDC sarcoma showed the most significant increase in Clusterin staining (**Figures 5C, D**). While the mean Clusterin DAB OD per GC was likely not striking enough to explain the increased clusterin observed globally in the LN-wide transcriptomic analysis, we found that there were significantly more GCs per area of the LN compared to related diseases and controls (**Figure 5E**), despite a significantly smaller size (**Figure 5F**). A model integrating regional Clusterin intensity with germinal-center density and size separated iMCD from non-iMCD LNs with an overall AUC of 0.94 (95% CI 0.87–1.00; permutation P = 0.001; **Figure 5G**). Discrimination was perfect against the lymphomas (DLBCL, FDC sarcoma; AUC 1.00) and high against sentinel reactive controls (0.97), but limited against SLE (0.69). Together, these analyses show that Clusterin is elevated in the GCs and MZs of iMCD patient LNs, and that these regions are more numerous in iMCD patient LNs than in related diseases.

**Figure 5:**
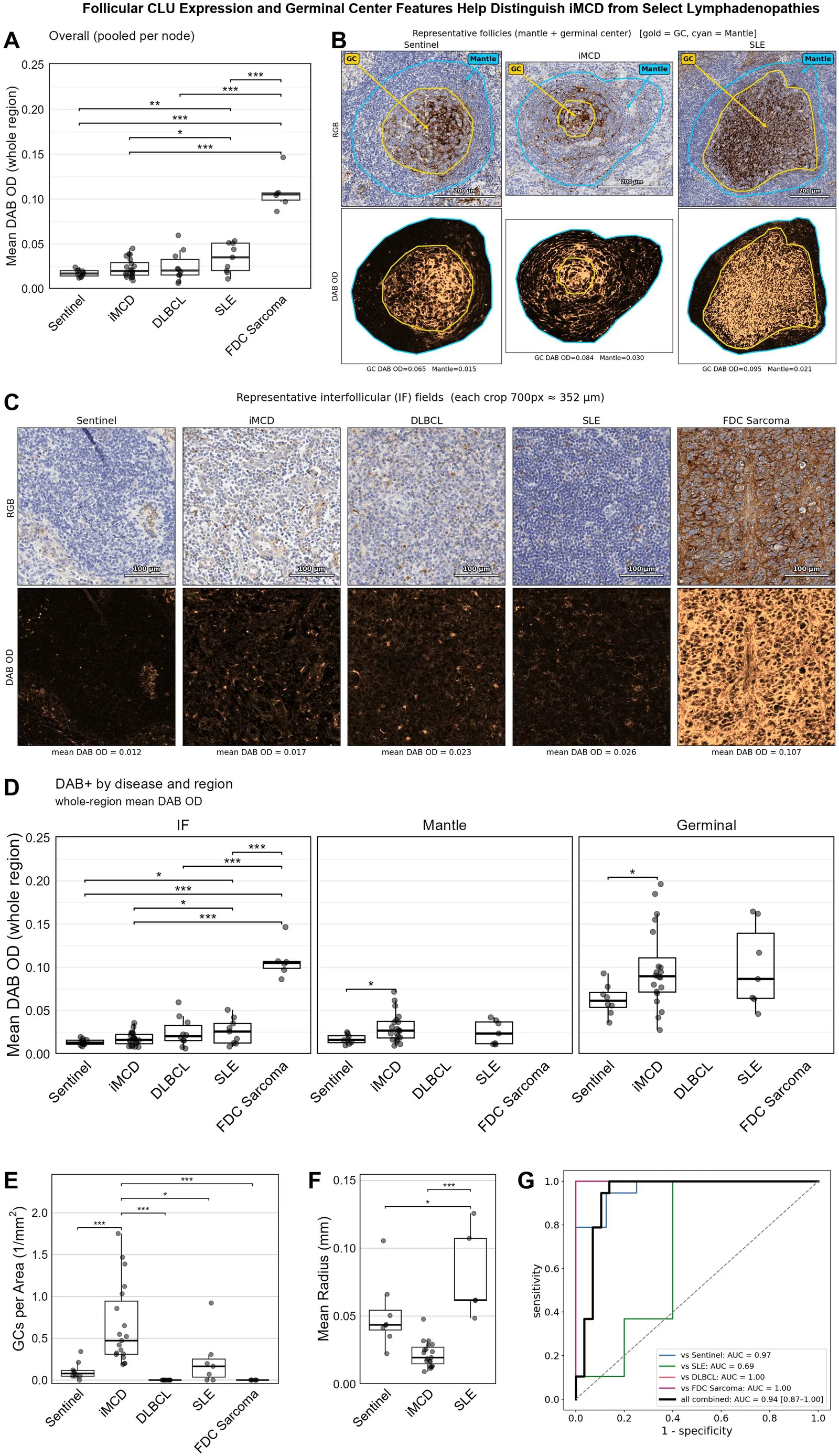
Follicular CLU expression and germinal center features help distinguish iMCD from select lymphadenopathies. Lymph nodes from sentinel (control), iMCD, DLBCL, SLE, and FDC Sarcoma patients were immunohistochemically stained for Clusterin protein using DAB and hematoxylin and imaged at 20X. Clusterin abundance was quantified as the mean DAB optical density (OD) within each region, computed from the color-deconvolved DAB channel. **(A)** Mean DAB OD across the whole lymph node region, pooled per node, for all conditions. **(B)** Representative follicles for sentinel, iMCD, and SLE lymph nodes, shown as RGB (top) and DAB OD (bottom), with the germinal center (gold) and mantle zone (cyan) outlined; mean DAB OD values for the GC and mantle are indicated. **(C)** Representative interfollicular (IF) fields for each condition, shown as the RGB image (top) and the corresponding DAB OD map (bottom); the mean DAB OD of each field is indicated below (each field ≈ 352 µm; scale bars, 100 µm). **(D)** Mean DAB OD by region (IF, mantle zone, and germinal center) for each condition. DLBCL and FDC Sarcoma had no identifiable germinal centers or mantle zones, and some sentinel lymph nodes had very few identifiable germinal centers. **(E)** The number of germinal centers per lymph node area (per mm²). **(F)** The mean radius of quantified germinal centers in each condition. **(G)** ROC analysis discriminating iMCD from non-Castleman lymphadenopathy. Coloured curves, the combined model’s separation of iMCD from each comparator (vs sentinel, AUC 0.97; vs SLE, 0.69; vs DLBCL and vs FDC sarcoma, both 1.00); black curve, the full combined model (compartment-resolved CLU intensity, GC density, GC size; AUC 0.94, 95% CI 0.87–1.00). **(A, D, E, F)** Statistical significance: ANOVA + Tukey test. P-value: *** <.001, ** <.01, * <.05.

## Discussion

This study is the first to employ multiple transcriptomic techniques across distinct subtypes of iMCD and various disease-control groups to characterize the LN transcriptome of these subtypes. We identified enrichment of several genes and gene networks previously described and consistent with disease biology, such as angiogenesis, cell proliferation, and various aspects of the immune response, including the IL-6-JAK-STAT pathway and mTORC1 signaling, which we previously discovered. Identifying these known pathways provides confidence in the ability to discover novel biologically plausible mechanisms. In addition to IL-6, several genes within the IL-6-JAK-STAT network were significantly upregulated (*SOCS3, OSMR, PIM1*). Even though the IL-6-JAK-STAT pathway is enriched in the serum of both IL-6-responsive and IL-6-refractory patients, IL-6 blockade is effective in approximately 35-50% of patients (4), prompting investigation into alternative mechanisms for IL-6-JAK-STAT activation in the absence of IL-6 (3). Recently, JAK inhibitors have been shown to be effective in alleviating symptoms in a subset of IL-6-refractory patients (34, 35).

After successfully validating a substantial number of upregulated genes and pathways identified in a discovery cohort of iMCD-TAFRO patients, we performed a targeted transcriptomic comparison between the differing iMCD subgroups using an orthogonal targeted gene expression assay. When looking for genes highly upregulated in each of the subtypes, we identified *SPP1*, *PRDM1*, *XBP1*, and *CLU. CLU* was the only gene that was upregulated in each iMCD clinical subtype, but not in any other disease group. Osteopontin (*SPP1*) regulates the inflammatory response and enhances the expression of VEGF family growth factor (36, 37). It was also found to be upregulated in a previous transcriptome-based study in MCD LN tissue, although clinical subset-level data were not available (13). Interestingly, one of the top therapeutic ‘reversers’ predicted from the enriched gene set signature in iMCD-TAFRO LN tissue was the VEGF inhibitor sorafenib, which, given these findings and the clinical observations of hypervascularization, warrants further investigation.

The upregulation of CLU across each subgroup was also interesting, as it serves as a follicular dendritic cell marker and mediates the clearance of apoptotic cells, thereby potentially preventing an apoptotic cell-induced autoimmune response (38). Because iMCD GCs undergo high GC B-cell turnover, CLU may be upregulated to facilitate debris clearance (39). High Clusterin levels could also inhibit dendritic cell death, increasing T-cell priming (40). FDCs internalize, recycle, and re-display complement-coated immune complexes via CR1 and CR2, presenting antigen to B-cells (41). Antigen presentation is a key driver in GC persistence and prolonged immune response (42). Clusterin binds terminal complement components and circulating immune complexes, forming soluble, non-lytic C5b-9, which could stabilize immune complexes in complement-rich follicles (41). Together, this suggests that *CLU* may play a role in GC persistence, B-cell activation, and plasma cell differentiation. Interestingly, *PRDM1* and *XBP1* are both transcription factors essential for the terminal differentiation of B cells into antibody-secreting plasma cells, suggesting a potential role for GC dysfunction in plasma cell differentiation.

In addition to differences in CLU expression between the GC and MZs of iMCD patients, we also observed significant differences in GC frequency and size compared with those in specific related disease controls. Taken together, this may suggest that the significant global increase in CLU expression observed in bulk transcriptomics is related to modestly increased CLU expression per unit area in the GC and MZ, as well as to substantially more GCs per unit area. Importantly, individual mantle zones and germinal centers were identified histologically, allowing CLU expression to be quantified within defined lymph node compartments. This compartment-level analysis demonstrated increased CLU expression in both mantle zones and germinal centers from iMCD lymph nodes compared with sentinel lymph nodes, suggesting that increased CLU expression is not explained solely by increased germinal center abundance. However, because CLU expression and germinal center abundance were assessed in the same histologic specimens, the current study cannot determine whether increased CLU expression contributes to germinal center expansion or represents a correlated feature of the iMCD lymph node microenvironment. A composite model incorporating CLU expression in the germinal center and mantle zone, GC density, and GC size demonstrated strong discriminatory performance for differentiating iMCD from selected related diseases (AUC 0.94), although validation in larger, independent cohorts will be needed to determine its broader diagnostic utility.

This study has several limitations. First, the cohort sizes are small, especially in the targeted gene expression cohort, reflecting the rarity of iMCD and the limited availability of well-characterized lymph node tissue from affected patients. The subtype-specific differences in gene expression should be interpreted in light of the limited sample size, as the number of iMCD-IPL (n=3) and iMCD-NOS (n=4) samples was small. Accordingly, these subtype-associated signatures should be viewed as exploratory observations that require validation in larger, independent cohorts. Second, cell-type deconvolution was performed using reference data derived from healthy donor lymph nodes. Given the architectural histology and immune activation characteristic of iMCD lymph nodes, this reference dataset may not fully capture disease-associated cellular states, which should be considered when interpreting inferred changes in plasma cells, monocytes, innate lymphoid cells, and other immune populations. Third, comparator groups included in this study were limited and do not encompass the full spectrum of lymphadenopathies that may enter the differential diagnosis of iMCD. Additional mimics, including IgG4-related disease, Rosai-Dorfman disease, angioimmunoblastic T-cell lymphoma, reactive follicular hyperplasia, HHV-8-associated MCD, and other autoimmune or inflammatory lymphadenopathies, were not included. Therefore, the ability of CLU expression to distinguish iMCD should be interpreted within the context of the comparator groups analyzed here. Larger validation studies, including a broader range of diagnostic mimics, will be needed to establish the broader specificity of CLU expression in iMCD. Finally, the LINCS-based drug repurposing analysis is entirely computational and should be considered hypothesis-generating. Although this approach identified biologically plausible candidates, including sirolimus, the predicted therapeutic relevance of these agents requires experimental and clinical validation before firm conclusions can be drawn.

In summary, we have conducted a comprehensive transcriptome investigation of iMCD LN tissue, with a particular focus on iMCD-TAFRO, the most severe subtype of iMCD. To extend those observations, we also used a targeted gene expression panel of immunology and inflammation-related genes in an expanded cohort of iMCD subtypes and other inflammatory disorders to identify shared and unique gene expression signatures in iMCD. Our findings support and validate previously identified gene markers in ‘omics-level investigations in multiple tissues in iMCD and provide additional functional candidates for further investigation. In addition, a composite model incorporating CLU expression in the germinal center and mantle zone, GC density, and GC size showed strong discriminatory performance for differentiating iMCD from selected related lymphadenopathies, supporting the potential value of integrating molecular and histomorphometric features. Together, these findings illuminate unique pathological mechanisms at play in iMCD lymph node tissue and provide a framework for future diagnostic and mechanistic studies.

## Data Availability

All Nanostring nCounter gene expression and bulk RNA sequencing data in this manuscript are available at GEO under the following GSE accession numbers: Nanostring nCounter: GSE336631 and bulk RNAseq: GSE337015. Publicly available data used in this paper can be found under the following GSE accession: GSE195477.
