# Supplementary material for: Clusterin expression and germinal center morphometry help distinguish idiopathic multicentric castleman disease from select lymphadenopathies
Source: Front Immunol. 2026 Sep 17;17:1872505. doi: 10.3389/fimmu.2026.1872505 (PMC13627302; doi:10.3389/fimmu.2026.1872505)

**Supplemental Figures**

**Figure S1:** PCA of top 500 variable genes in iMCD-TAFRO vs control dataset

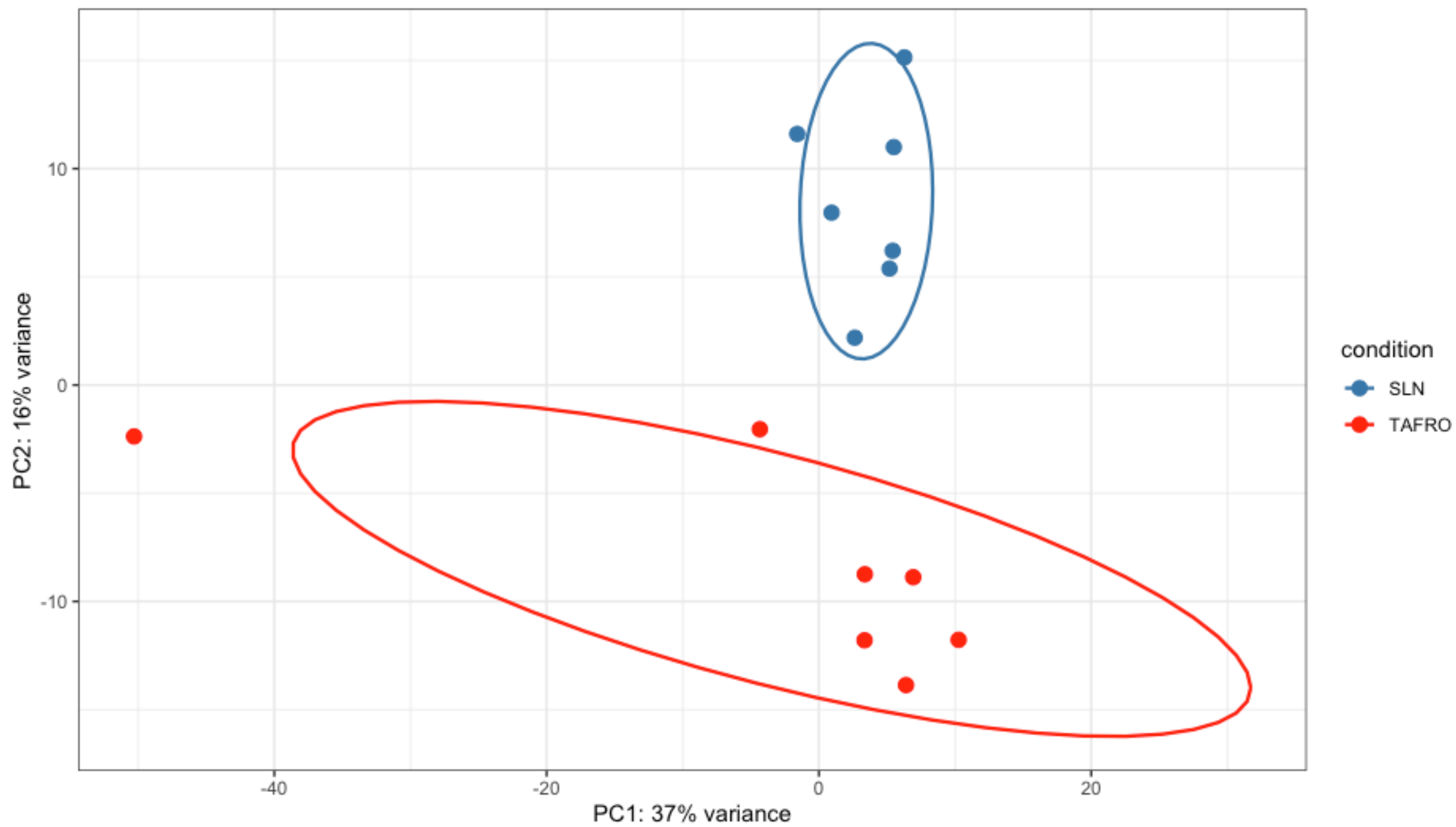

**Figure S2A:** Top loadings for PC1 in iMCD-TAFRO versus sentinel control samples

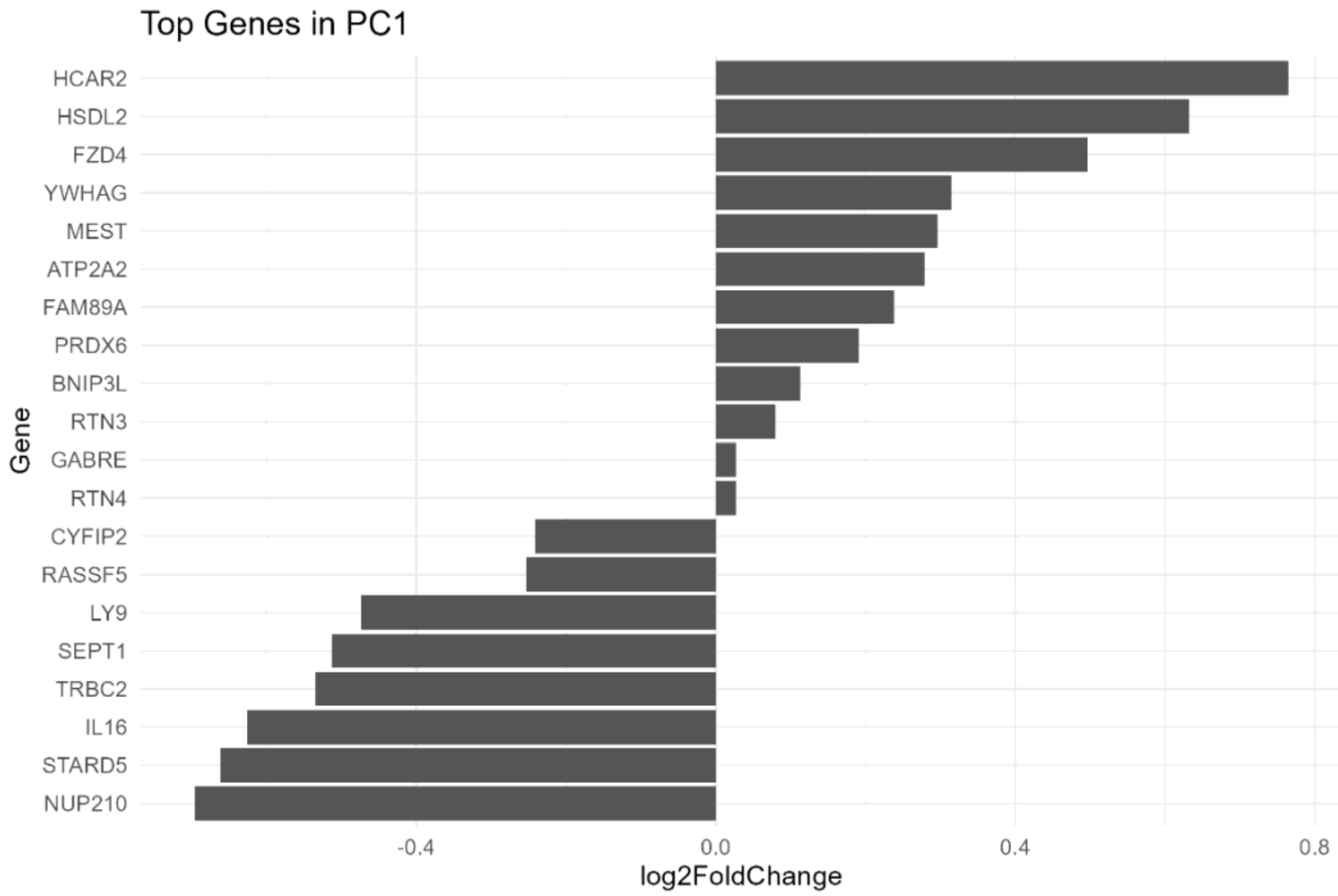


**Figure S2B:** Top loadings for PC2 in iMCD-TAFRO and control samples

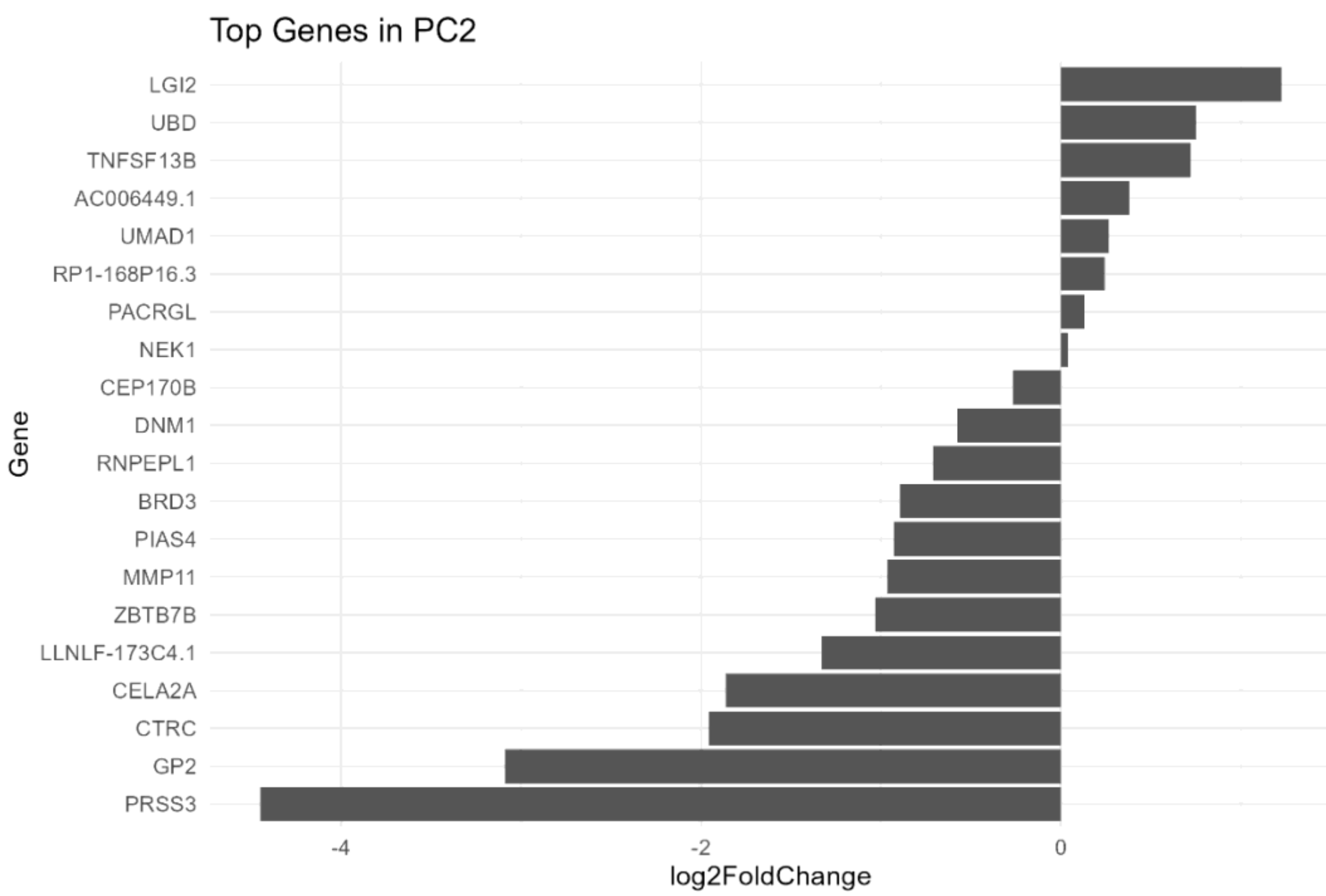

**Figure S3:** Assessment of batch (source of samples) correction in the TAFRO versus control validation study using limma

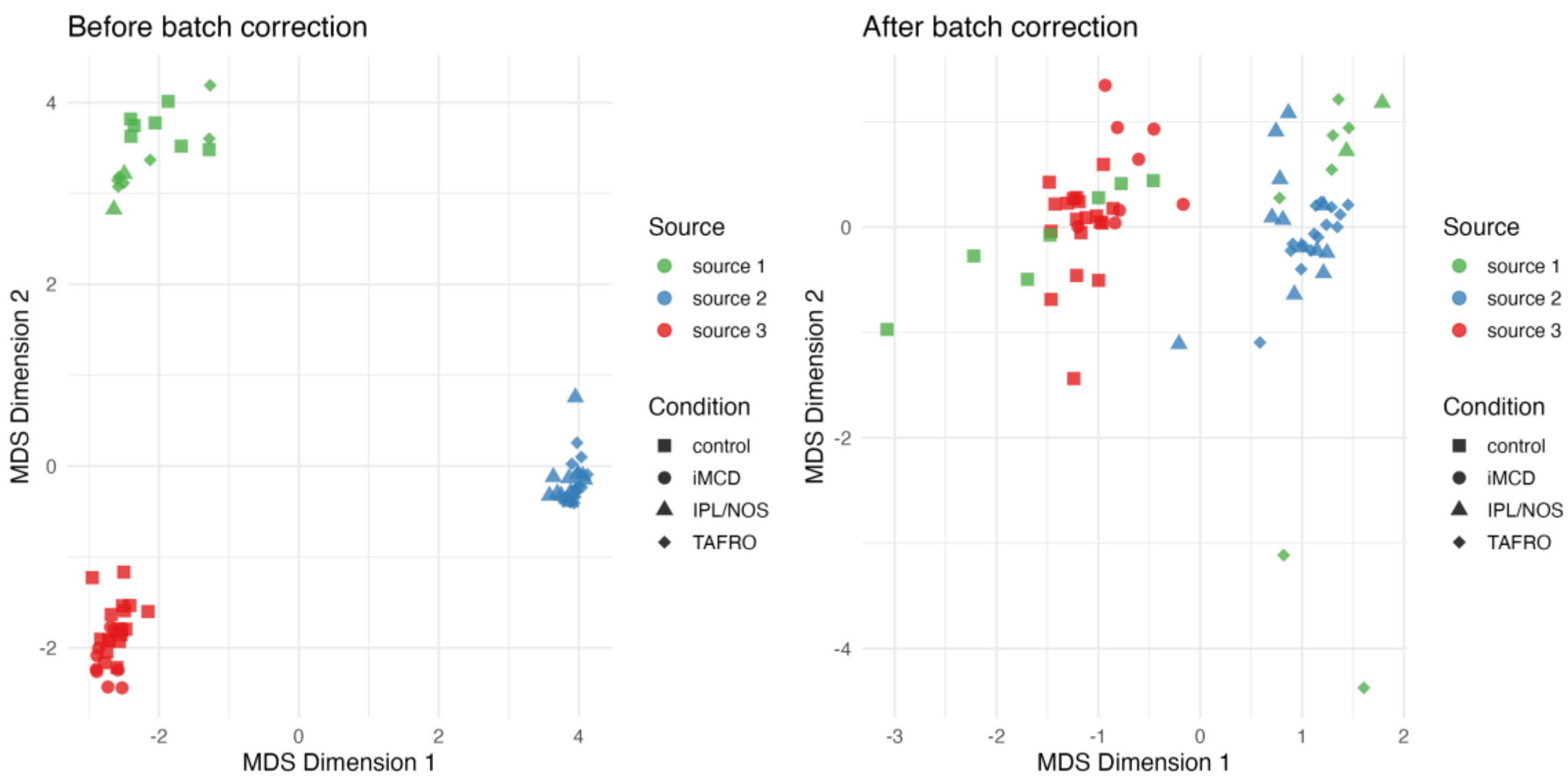

**Figure S4:** Correlation of log2 fold changes between the discovery cohort and the validation cohort for iMCD-TAFRO versus control samples.

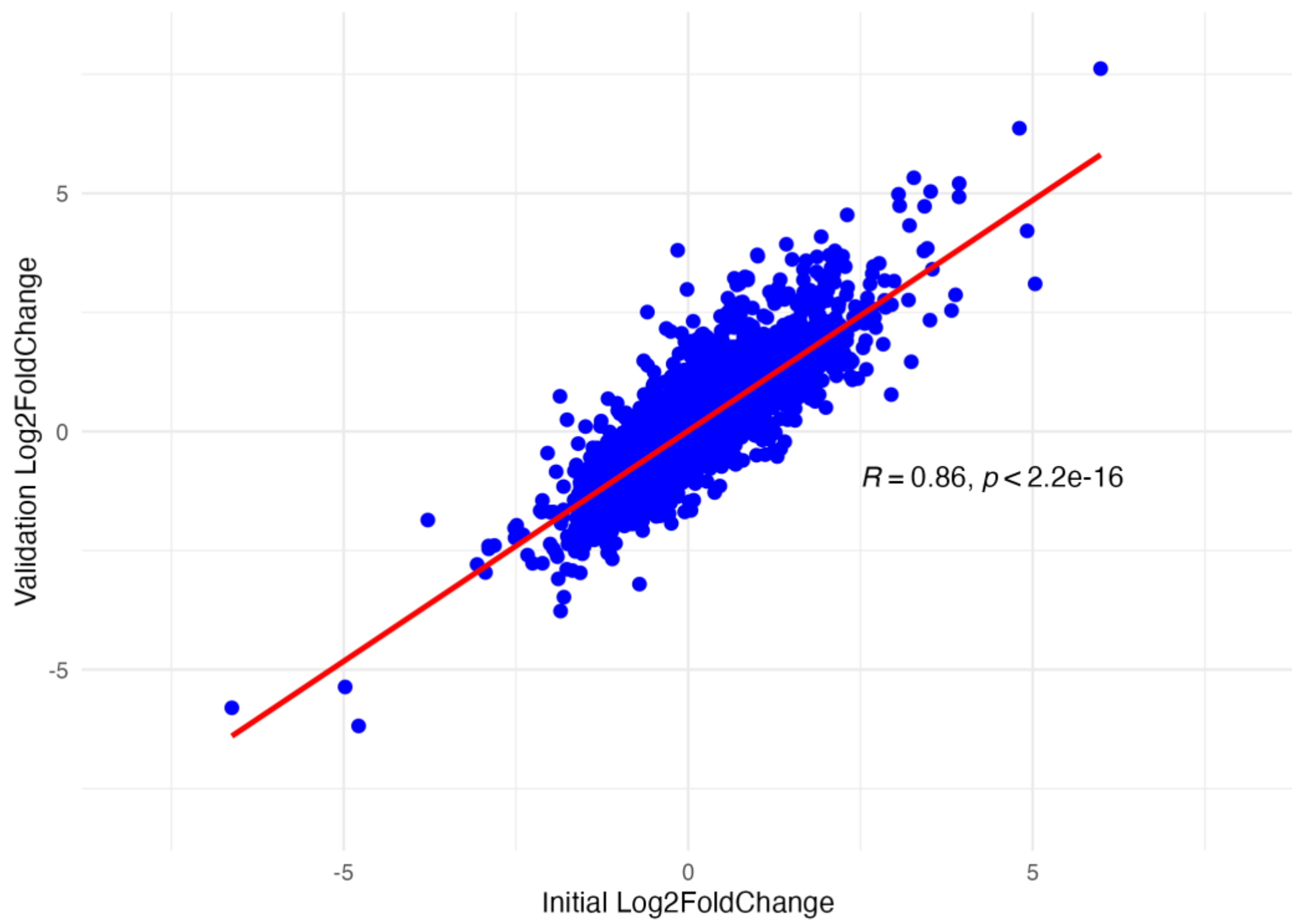

**Figure S5:** Correlation of gene expression results between Nanostring nCounter and bulk RNA-seq analyses.

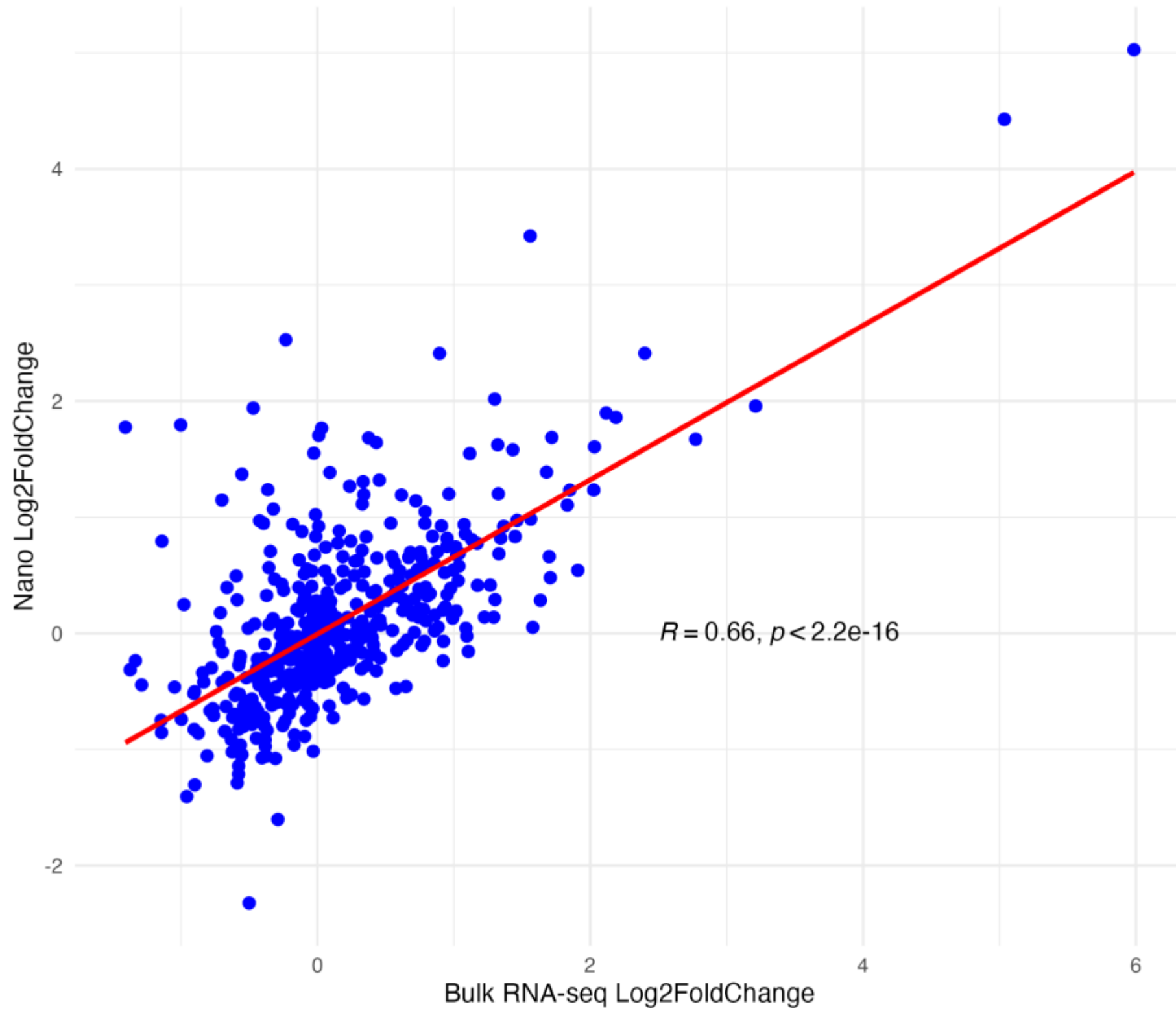

Supplement: Supplementary file 1 [file DataSheet1.pdf]
